# Metabolic cost in healthy fit older adults and young adults during overground and treadmill walking

**DOI:** 10.1007/s00421-021-04740-2

**Published:** 2021-06-21

**Authors:** Sauvik Das Gupta, Maarten Bobbert, Herre Faber, Dinant Kistemaker

**Affiliations:** 1 Department of Human Movement Sciences, Faculty of Behavioural and Movement Sciences, Vrije Universiteit Amsterdam, Amsterdam Movement Sciences, Amsterdam, The Netherlands; 2 Human Movement Biomechanics Research Group, Department of Movement Sciences, Biomedical Sciences Group, KU Leuven, Leuven, Belgium; 3 Faculty of Health, Nutrition and Sports, The Hague University for Professional Education, The Hague, The Netherlands

**Keywords:** Healthy aging, Gait, Energetic cost, Preferred walking speed, Gerontology

## Abstract

**Purpose:**

The purpose of this study was to determine whether net metabolic cost of walking is affected by age per se.

**Methods:**

We selected 10 healthy, active older adults (mean age 75 years) and 10 young adults (mean age 26 years), and determined their preferred overground walking speed. On the same day, in a morning and afternoon session, we had them walk at that speed overground and on a treadmill while we measured oxygen consumption rate. From the latter we subtracted the rate in sitting and calculated net metabolic cost.

**Results:**

Anthropometrics were not different between the groups nor was preferred walking speed (1.27 m s^−1^ both groups). There was no difference in net metabolic cost of overground walking between older and young adults (e.g., in the morning 2.64 and 2.56 J kg^−1^ m^−1^, respectively, *p* > 0.05). In the morning session, net metabolic cost of walking was higher on the treadmill than overground in our older adults by 0.6 J kg^−1^ m^−1^ (*p* < 0.05), but not in young adults.

**Conclusion:**

First, there is no effect of age per se on metabolic cost of overground walking. Second, older adults tend to have higher metabolic cost of walking on a treadmill than walking overground at preferred speed, and adaptation may take a long time. The commonly reported age-related elevation of metabolic cost of walking may be due to confounding factors causing preferred walking speed to be lower in older adults, and/or due to older adults reacting differently to treadmill walking than young adults.

## Introduction

The ability to walk is important for people to participate in society. Healthy young people walk seemingly without physical effort, but when people get older, walking can become a challenge. An important variable in studying walking is the metabolic cost of walking (MCoW), defined as the metabolic energy expended per kilogram body mass per meter traveled. MCoW is typically calculated from measured oxygen consumption, carbon dioxide production and walking speed (Zarrugh and Radcliffe 1978), and can be expressed in terms of gross cost of walking (GCoW) and in terms of net cost of walking (NCoW). GCoW relates to the total amount of metabolic energy consumed, whereas NCoW, calculated by subtracting from the metabolic rate during walking the resting metabolic rate, captures the amount of metabolic energy expended due to walking per se (i.e., due to walking-related cross-bridge cycling and Ca^2+^ pumping within muscles). MCoW has been studied across all age-groups in humans, from children to the elderly. Typically, healthy young adults (YA) walking at their preferred walking speed (PWS) exhibit a GCoW of about 3.4 J kg^−1^ m^−1^ and a NCoW of about 2.4 J kg^−1^ m^−1^. When published results on differences between YA and older adults (OA) for MCoW are summarized, OA have a statistically significantly elevated MCoW compared to YA; the pooled mean GCoW was ~ 0.3 J kg^−1^ m^−1^ (*d* = 0.65) higher in OA than in YA, and the pooled mean NCoW was ~ 0.4 J kg^−1^ m^−1^ (*d* = 1.00) higher in OA than in YA (Das Gupta et al. 2019).

In the literature, the increase in MCoW in OA has been attributed to age-induced physiological changes in muscles and the cardiovascular system (Gaesser et al. 2018), changes in gait coordination (Donelan et al. 2001), and/or higher co-activation reflected in Electromyography (EMG) activity (Peterson and Martin 2010). However, in our meta-analysis (Das Gupta et al. 2019) we indicated several potential confounding factors that made it difficult to judge whether the difference in MCoW found in the literature is a direct effect of age.

**Fig. 1:**
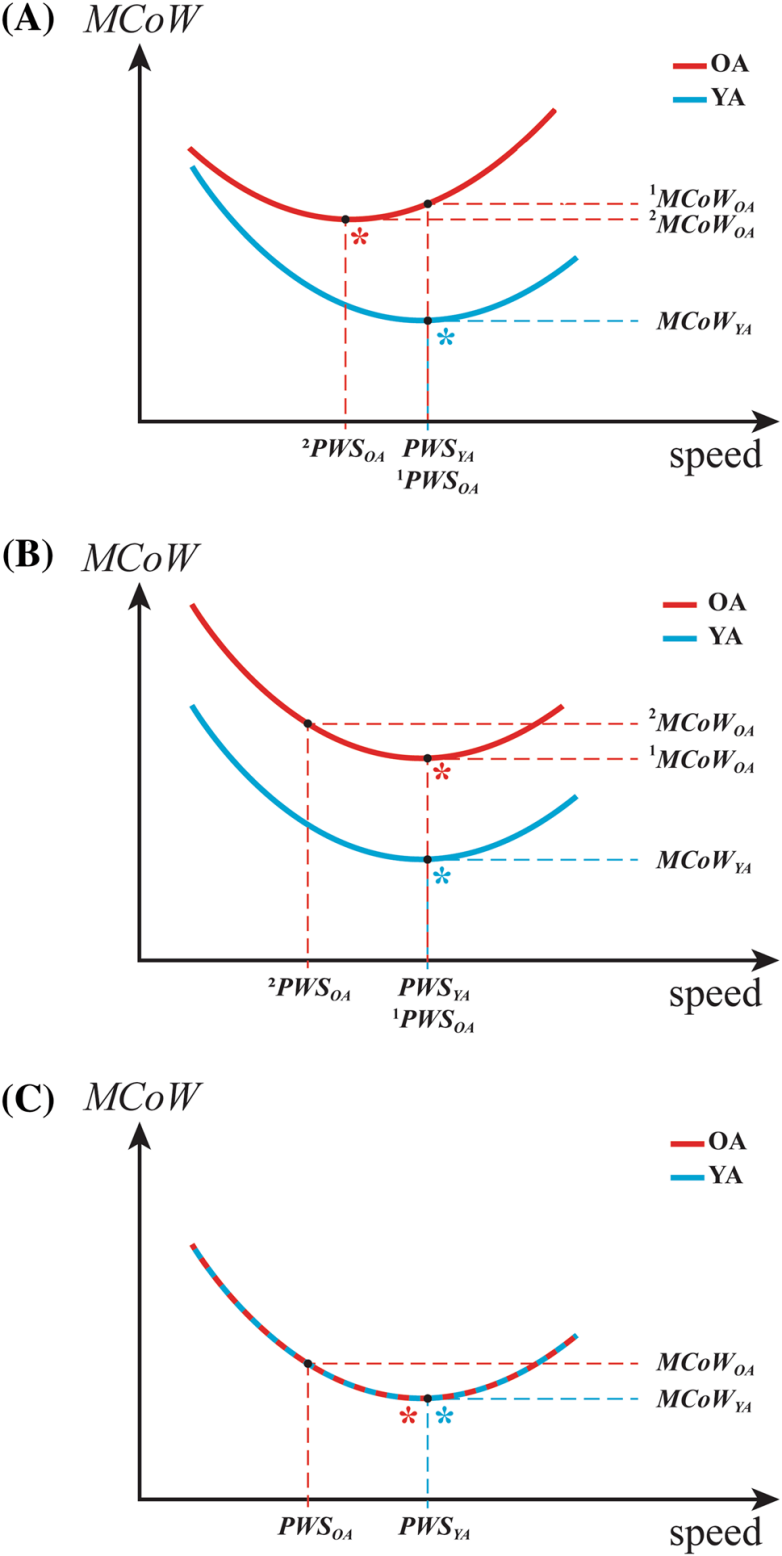
Possible scenarios underlying an elevated metabolic cost of walking (MCoW) in older adults (OA) compared to Young Adults (YA). At a given MCoW-curve, the asterisk (*) indicates the optimal walking speed, i.e., the speed at which MCoW is minimal. In scenario A the MCoW curve is shifted leftward and upward in OA compared to YA, in scenario B it is only shifted upward, and in scenario C it is not shifted at all. In scenario A, two cases are shown leading to an elevated MCoW in OA: in the first case Preferred Walking Speed (PWS) of OA is sub-optimal (^1^PWS_OA_) yet similar to that of YA resulting in ^1^MCoW_OA_, and in the second case ^2^PWS_OA_ is optimal and resulting in ^2^MCoW_OA_. In scenario B, MCoW is elevated in OA, both when OA are selecting a PWS that is sub-optimal (^2^PWS_OA_) and when they select a PWS that is optimal/similar to that of YA (^1^PWS_OA_). In scenario C, there is no (statistically) significant difference in MCoW curves and only if OA select a lower (sub-optimal) PWS than YA, their MCoW would be higher. Selecting a (for NCoW) sub-optimal PWS in OA may, for example, be caused by increased stability demands or insufficient familiarization time

A first possible confounder is PWS. In two studies in which overground walking was studied, OA were reported to have a higher MCoW than YA (Waters et al. 1983, 1988), and also had a lower PWS than YA. It is generally known from the literature that MCoW depends on walking speed (Ralston 1958; Zarrugh et al. 1974), and that YA prefer to walk at the speed at which MCoW is minimized (Ralston 1958). Due to an age-related reduction of fitness, or disease, OA may prefer a walking speed which is lower than the speed at which MCoW is minimized (Sanseverino et al. 2018). It cannot be decided to what extent the difference in MCoW between OA and YA in the two studies on overground walking is due to age per se and to what extent it is due to a difference in walking speed; potential scenarios for a confounding effect of walking speed are presented in Fig. 1A, B.

A second possible confounder in studying age effects on MCoW is the use of a treadmill. Possibly to avoid a confounding effect of PWS in overground walking, all other studies that we found on the difference in MCoW between OA and YA have been on treadmill walking at fixed speeds (Dean et al. 2007; Floreani et al. 2018; Jones et al. 2009; Martin et al. 1992). However, OA have a higher metabolic requirement at PWS on a treadmill than overground (Parvataneni et al. 2009). It has been suggested that OA need more familiarization time than YA to restore ‘normal’ walking kinematics (Schellenbach et al. 2010; Wass et al. 2005), and thus require more time to arrive at their steady state MCoW. It is possible that the investigators may not have provided OA with enough time to familiarize to treadmill walking per walking condition [see: (Das Gupta et al. 2019)], which could have resulted in an elevated MCoW compared to YA. Hence, if one is interested in the ecologically valid difference in MCoW between OA and YA in overground walking, the use of a treadmill may itself be a confounder.

The third possible confounder is physical fitness. It is known that due to aging there is a general decline of the physical activity and fitness, which in turn may affect PWS (Haveman-Nies et al. 1996; Jones et al. 2009; Malatesta et al. 2003; Martin et al. 1992; Pincheira et al. 2017; Waters et al. 1983, 1988). A reduction in PWS of OA due to a decline in fitness may lead to an elevated MCoW for OA compared to their fitter YA counterparts, even if the (U-shaped) relationship between MCoW and walking speed itself is the same for YA and OA; this possible scenario is shown schematically in Fig. 1C.

The fourth possible confounder is a difference in anthropometrics. MCoW is commonly reported in mass-normalized values, which implicitly assumes that all participants have the same proportion of muscle mass to body mass. However, particularly in sedentary elderly this proportion might be smaller than in active young participants. It has been suggested in the literature that normalizing physiological variables to body mass may substantially influence the (statistical) outcomes; it may actually introduce a methodological bias and lead to inflated type I errors (Packard and Boardman 1999).

The purpose of the present study was to determine whether net metabolic cost of walking is affected by age per se. We set out to measure MCoW in YA and OA while attempting to control the possible confounders mentioned above. To end up with anthropometrically matched groups of physically active, fit and healthy OA and YA, we used strict inclusion criteria for potential participants. To investigate the potential influence of mass-normalization of physiological variables [see: (Packard and Boardman 1999)], we performed an ANCOVA. We determined overground PWS, and we measured GCoW and NCoW at each individual’s PWS both overground and on a treadmill, and both in a morning session and in an afternoon session on the same day. In the afternoon session, we additionally measured kinematics, kinetics and EMG, but in this study we report only GCoW and NCoW. We focused on the following questions: (1) is MCoW in overground walking higher in OA than in YA? (2) Is MCoW in treadmill walking higher than in overground walking in OA and in YA? (3) Is MCoW in treadmill walking higher in OA than in YA?

## Methods

### Characteristics of participants and Ethics statement

For this study, we recruited 10 healthy OA (mean age 75.3 ± 6.3 years, five males and five females) and 10 healthy YA (mean age 25.5 ± 3.4 years, four males and six females). The sample size per group was estimated from published studies as also done in Horiuchi et al. (2015) and comparable to that in other studies (Dean et al. 2007; Horiuchi et al. 2015; Hortobágyi et al. 2011; Malatesta et al. 2003, 2004; Ortega and Farley 2007, 2015; Ortega et al. 2008; Peterson and Martin 2010; Pincheira et al. 2017). We excluded participants who had chronic heart disease, diabetes, past surgeries or prosthesis on the lower limbs, or neuromuscular disabilities, or had experienced a fall in the past 6 months. In soliciting potential participants, we stressed that they should be fit and physically active, carrying out their normal day-to-day activities without any assistance. We ended up accepting only those participants who met these criteria. None of the participants was actively involved in any kind of special strength or endurance training. The inclusion/exclusion criteria were exactly similar for both YA and OA and thus we believe that they have not introduced new confounding factors. We asked all our participants to specify their general day-to-day activity patterns and in hindsight feel safe to say that there was no selection bias between our YA and OA. All participants signed a written informed consent. We measured standard anthropometrics; body mass, height, waist and hip circumferences and lower limb lengths up to malleolus and foot from the greater trochanter. Body adiposity index (BAI) was calculated according to the following equation (Bergman et al. 2011):

1 $${\text{BAI}}(\% ) = \left( {100 \times c_{{{\text{hip}}}} /h^{{3/2}} } \right) - 18,$$

where *c*_hip_ is the circumference measured at the height of the hip and *h* is the body height, both in *m*.

The ethical review committee of the Faculty of Behavioural and Movement Sciences of the Vrije Universiteit Amsterdam approved the experimental protocol.

### Protocol

#### Pre-experiment factors

Detailed instructions regarding diet and exercise were given to all YA and OA participants. In short, participants were to have a light meal for both their breakfast and lunch (i.e., moderate intake of carbohydrates and fats, minimal intake of protein and fiber rich foods; food intake was listed). They were also instructed to refrain from consuming any caffeine or alcohol-containing products and from smoking tobacco until the experiments were finished. To negate the thermic effect of food, we ensured that there was an interval of at least 2 h between their meals and measurement of any metabolic data. In total, there was at least a 3-h interval between the two experimental sessions. Within each session, there were at least 20 min between the overground trial and the treadmill trial.

#### Preparation

The participants were fitted with a portable Cosmed K4b2 breath-by-breath indirect calorimetry system (Cosmed, Rome, Italy) and a face-mask to measure rates of oxygen consumption and carbon dioxide production. The Cosmed K4b2 was calibrated before the start of each experimental session according to the manufacturer’s guidelines.

#### Morning and afternoon session

We had our participants walk in a morning session and in an afternoon session on the same day. We chose to have two sessions in order to allow our OA to familiarize themselves with walking in our laboratory, and in particular on our treadmill. This was based on studies showing that OA need more time (~ 15 min) to arrive at kinematics that are similar to those observed during overground walking (Schellenbach et al. 2010; Wass et al. 2005) than YA. The morning session consisted of two trials; one overground trial (9 min) and one treadmill trial (15 min). The afternoon overground trial was of similar duration as the morning overground trial. The afternoon treadmill trial consisted of 9 min walking and not 15 min like the morning session, because OA had indicated in pilot studies that a 15 min treadmill trial at the end of the day was too tiring.

**Fig. 2:**
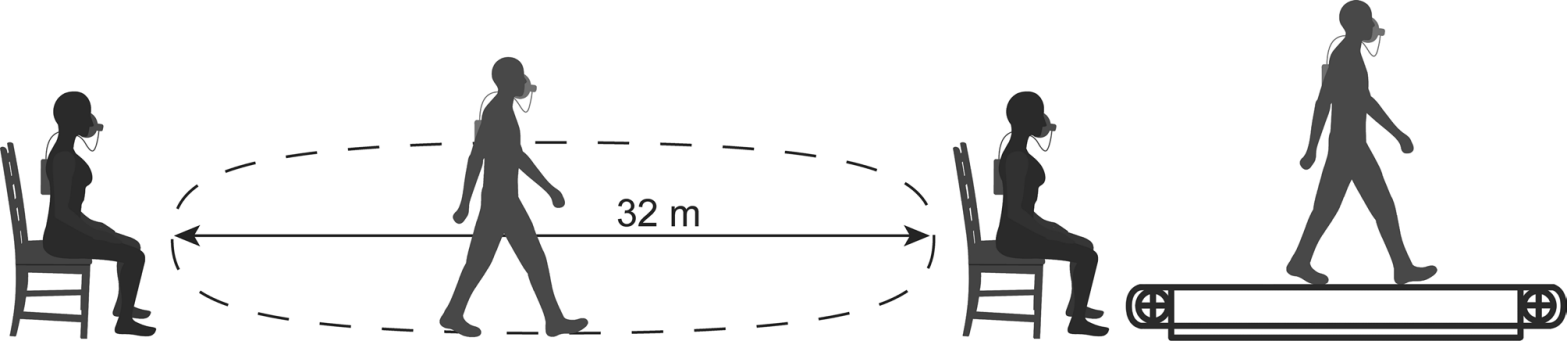
Schematic overview of the energy expenditure measurements during one session. Resting Metabolic Rate was measured while the participants were seated for 5 min on a chair as shown above, both before the overground walking trial and before the treadmill trial. Metabolic Cost of Walking was measured for both young and elderly adults in two separate sessions, each involving an overground trial and a treadmill trial. During the overground trial participants walked continuously along the schematically drawn oval track with 32 m straights interconnected by two half-circles of 4 m radius. During the treadmill trial participants walked on a treadmill at their imposed overground PWS

Prior to every walking trial, resting metabolic rate (RMR) was measured while the participants were seated in a chair for 5 min in a relaxed state (see: Fig. 2). In several other studies, RMR was measured during standing. The RMR measured during standing is about 1.16 times the RMR measured during resting; the additional metabolic cost is attributed to activation of leg and trunk muscles to maintain an upright posture, support the body weight and to balance (Weyand et al. 2009). The same cost is also involved in walking and in our opinion is part of the cost of walking. Hence, we feel that when using NCoW to estimate the metabolic cost attributed to walking itself, it is more meaningful to subtract RMR in a non-standing posture to estimate NCoW.

All participants then walked continuously (without any pause) at their preferred speeds inside a large indoor laboratory along an oval track with 32 m straights interconnected by two half-circles of 4 m radius (Fig. 2). They did so for at least 5 min and then completed at least three full laps during which oxygen consumption rate was recorded. GCoW (in J kg^−1^ m^−1^) was calculated from the oxygen consumption rate using Lusk’s equation (Lusk 1923) and dividing by PWS:

2 $${\text{GCoW}} = (15962 + 5155 \cdot {\text{RER}}) \cdot \dot{V}{\text{O}}_{2} \cdot {\text{PWS}}^{{ - 1}} ,$$

where RER is the respiratory exchange ratio (dimensionless), $\dot{V}{\text{O}}_{2}$ is oxygen consumption rate (in l kg^−1^ s^−1^), and PWS was the average walking speed during the last three laps (in m s^−1^). In order to establish PWS participants were asked in the morning overground trial to walk at the speed they would normally walk while going from their home to a supermarket. NCoW was obtained by inserting net $\dot{V}{\text{O}}_{2}$, i.e., the difference in $\dot{V}{\text{O}}_{2}$ between walking and sitting.

Following the overground trial, all participants walked for 15 min on a dual-belt motorized treadmill (ForceLink BV, Culemborg, the Netherlands) while oxygen consumption rate was measured (Fig. 2). Treadmill speed was gradually increased over the first 2–3 min to the individual’s overground PWS, and then kept constant. At the end of the trial, the treadmill speed was gradually reduced to zero and then oxygen consumption rate measurement was stopped. The last 30 s of data were always discarded and the oxygen consumption rate of the preceding last three full minutes was used to calculate MCoW for all our participants and all the treadmill trials.

In the afternoon session, we first instrumented the participants with kinematic markers and EMG-electrodes, and then repeated the overground walking and treadmill trials while we measured not only oxygen consumption rate, but also kinematics, EMG and ground reaction forces. Note, however, that in this paper, we only focus and report on MCoW.

### Statistical tests

Before analyzing differences between OA and YA in anthropometric parameters and PWS, we first used a Shapiro–Wilk test to check for normality. We then used a Levene’s test to check for the equality of variances. In addition, Quantile–Quantile plots, boxplots and violin plots were used to visually check for normality. To analyze between-group differences in anthropometric parameters and PWS we used the parametric two-sided independent samples Student’s *t* test.

Before embarking on a full-blown statistical analysis of results of NCoW, we averaged GCoW and NCoW time histories over participants and graphically displayed means and Standard Error of the Mean (SEM) as a function of time for the last 4 min of the trials. From these graphs, it can already be deduced which differences among groups are not statistically significant and which differences might be significant. We then performed an ANCOVA of NCoW normalized for distance only with body mass of the participants to check whether the outcome of our study could have been inadvertently affected by mass normalization (Packard and Boardman 1999). To analyze both between-group and within-subject differences in NCoW between our YA and OA participants, we performed a Mixed ANOVA analysis with three levels: group (OA versus YA), ‘surface’ (overground versus treadmill) and session (morning versus afternoon). We further analyzed statistically significant main effects and interaction effects using Post hoc tests with Tukey’s correction for non-repeated measures tests and Bonferroni’s correction for repeated measures tests. As will be explained in the discussion, we also tested a few differences using simple *t* tests without correction to see whether false negatives had occurred.

The effect sizes (Hedge’s *G*) for the comparisons between age, anthropometric parameters and PWS were calculated as the mean values for YA subtracted from the mean values for OA divided by the pooled standard deviation of both the groups. The effect sizes for both the within-subject and between-group comparisons on NCoW from the Mixed ANOVA analysis will be reported as partial eta-squared ($\eta_\text{p} ^{2}$). Effect sizes of the post-hoc comparisons and those of the separate unpaired and paired Student’s *t* tests as mentioned in the Discussion section are reported as Cohen’s *d*. The open-source software JASP (version 0.13.0.0) was used for all the statistical tests. The default value of *α* = 0.05 was chosen as the level of statistical significance.

## Results

We found no deviations from normality or equality of variances for any of the anthropometric parameters or PWS, with one exception: for Body mass index (BMI) the assumption of equality of variances was violated. Hence, for testing differences in BMI we resorted to the parametric two-sided independent samples Welch’s *t* test.

### Age, walking speed and anthropometrics

Table 1 lists the age, anthropometric parameters and PWS of the participants as mean ± SD (standard deviation). Other than the obvious difference in age, there were no statistically significant differences between YA and OA in anthropometric parameters or PWS (*p* > 0.05); PWS was 1.27 m s^−1^ both in OA and YA.

**Table 1:** Age, anthropometric parameters and PWS of YA and OA

	YA	OA	Effect size
Age (years)	25.5 ± 3.4	75.3 ± 6.3	9.4
Body mass (kg)	62.4 ± 12.5	63.3 ± 7.3	0.1
Height (m)	1.63 ± 0.10	1.65 ± 0.09	0.2
BMI (kg/m^2^)	23.6 ± 4.5	23.2 ± 2.3	− 0.1
BAI (%)	28.7 ± 5.9	28.0 ± 4.2	− 0.1
W/H ratio	0.5 ± 0.1	0.5 ± 0.1	0
Lower limb length up to malleolus (m)	0.79 ± 0.05	0.80 ± 0.05	0.2
Lower limb length up to foot (m)	0.84 ± 0.06	0.86 ± 0.06	0.3
Overground PWS (m s^−1^)	1.3 ± 0.2	1.3 ± 0.1	0

*BMI* body mass index, *BAI* body adiposity index, *W/H* waist-to-height

### Metabolic cost of walking

**Fig. 3:**
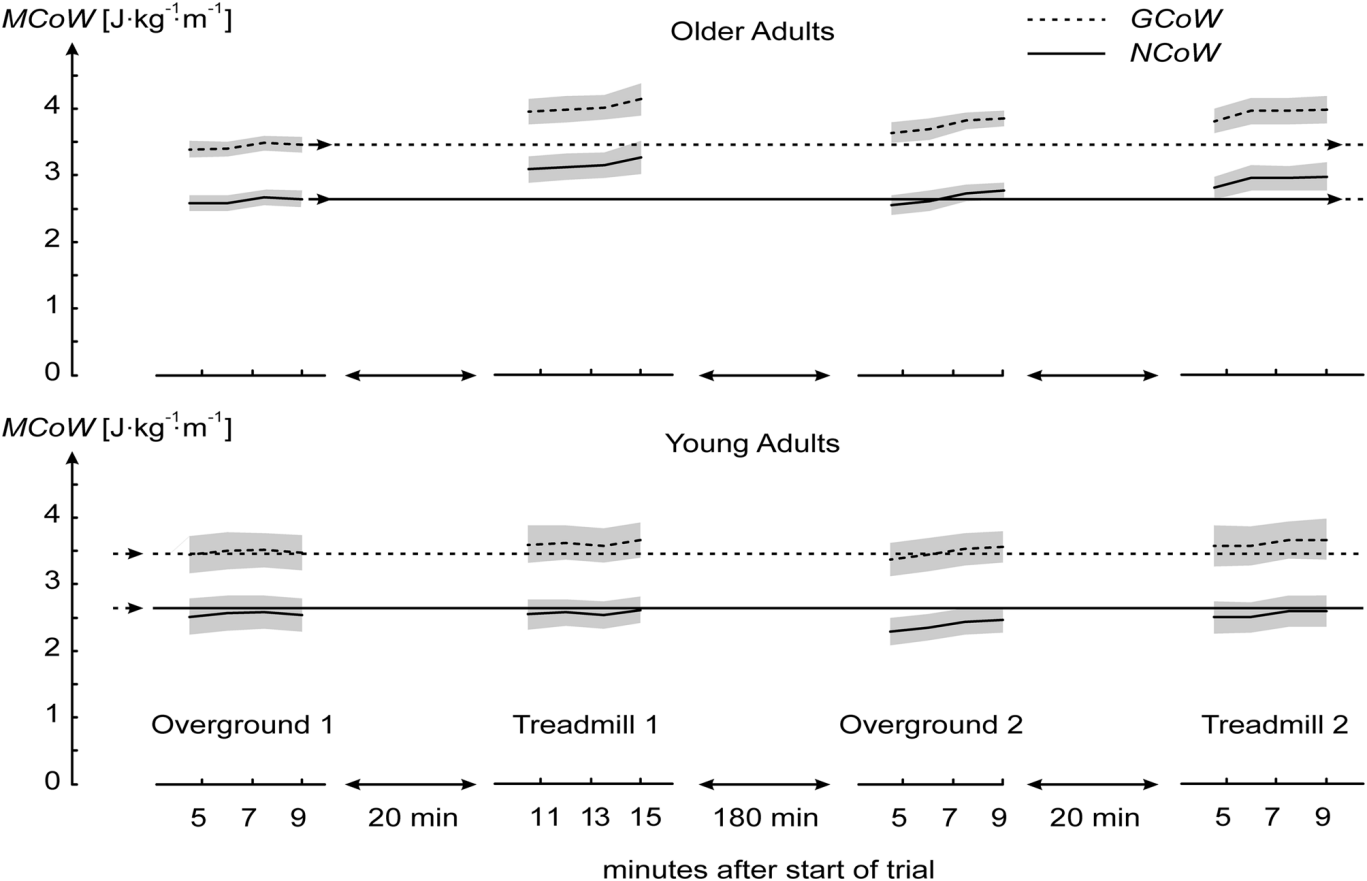
Overview of metabolic cost results. Means and SEM (standard error of mean, gray area) of Gross and Net Cost of Walking (GCoW and NCoW, respectively) have been plotted for all trials. Just for easy reference, long horizontal lines in the top diagrams and bottom diagrams are plotted at the values attained by older adults at the end of the first overground walking trial

Figure 3 provides a helicopter view of means and SEM (standard error of mean, gray area) of GCoW and NCoW for YA and OA for all trials. The long horizontal lines in the top diagrams (OA) and bottom diagrams (YA) are for easy reference; they are plotted at the values attained by OA at the end of the first overground walking trial. Just as a first indication, for a difference between means in OA and YA to be statistically significant, it should be bigger than about twice the pooled SEM. In YA, GCoW and NCoW seemed to reach a steady state value that was the same on treadmill and overground. In OA, however, GCoW and NCoW tended to increase during the treadmill trials (even after 10 min in the morning treadmill trial) and seemed to reach values that were higher on the treadmill than overground. This increase in GCoW and NCoW after 10 min in the morning treadmill trial occurred specifically in three OA, which was due to a slow increase in the rate of oxygen consumption for all of them and additionally by an increase in RER in one of these three OA. The slow increase in the rate of oxygen consumption could indicate that muscle fibers become less efficient and it could be a sign of fatigue (Barclay 1996; Jones et al. 2011; Woledge 1998). In addition, it could also be that fatigue led these OA to walk with a different gait pattern, perhaps with more muscle coactivation, which in turn led to the increase in MCoW. As a matter of fact, indeed two out of these three OA felt it quite hard to complete the 15 min treadmill trial and reported feeling fatigued after the trial. All these three OA were also at least 80 years old or above, possibly indicating that such a manifestation of fatigue was seen only in our octogenarians.

As our YA and OA groups were anthropometrically matched (see: Table 1), it was unlikely that differences in anthropometrics substantially influenced our outcomes. The results of the ANCOVA confirmed this; there was no statistical effect of body mass normalization.

Our mixed ANOVA detected no problems with data homogeneity. It yielded only two statistically significant effects, a main effect of ‘surface’ (*p* = 0.04, $\eta ^{2}_{{\text{p}}}$ = 0.21) and a combined interaction effect of group*surface*session (*p* = 0.02, $\eta ^{2}_{{\text{p}}}$ = 0.27). Overall there were no group effects (YA vs OA, *p* = 0.16, $\eta ^{2}_{{\text{p}}}$ = 0.11); there were no violations of data homogeneity.

Our first question was whether OA had a higher NCoW than YA during overground walking. During the morning overground trial, NCoW was 2.64 ± 0.38 J kg^−1^ m^−1^ in OA and 2.56 ± 0.79 J kg^−1^ m^−1^ in YA, a difference of only 0.08 J kg^−1^ m^−1^. During the afternoon overground trial the difference was 0.3 J kg^−1^ m^−1^. According to the Mixed ANOVA results, these differences were not statistically significant (*p* > 0.05) after post-hoc corrections.

Our second question was whether NCoW in treadmill walking was higher than in overground walking in OA and in YA. Post hoc tests revealed that NCoW was overall higher on the treadmill than overground by 0.24 J kg^−1^ m^−1^ (*p* = 0.04) with a medium effect size (Cohen’s *d* = 0.48). Specifically, NCoW was higher on the treadmill than overground in OA in the morning session by 0.61 J kg^−1^ m^−1^ (*p* = 0.03). This was already the case after 5 min of treadmill walking, when none of the OA reported any fatigue.

Our third question was whether OA had a higher NCoW than YA during treadmill walking. During the last three full minutes of the morning treadmill trial, NCoW in OA was 3.25 ± 0.68 J kg^−1^ m^−1^ and 2.59 ± 0.6 J kg^−1^ m^−1^ in YA, a difference of 0.66 J kg^−1^ m^−1^. During the afternoon treadmill trial the difference was smaller, 0.36 J kg^−1^ m^−1^. According to the mixed ANOVA, there was no group effect and these differences were also not statistically significant (*p* > 0.05) after post-hoc corrections. Note, however, that the afternoon treadmill trial was shorter than that in the morning; NCoW continued to increase until the end of the morning treadmill trial, and might have increased further in the afternoon trial if that trial had lasted longer.

In Fig. 3 it can be seen that the curves of GCoW and NCoW in the afternoon are further apart than those in the morning. This is mostly because RMR was higher in the afternoon by 7.3% in YA and by as much as 25% in OA. For YA, RMR was 77 ± 19.4 J kg^−1^ min^−1^ in the morning session and 82.6 ± 14.7 J kg^−1^ min^−1^ in the afternoon session. For OA, RMR was 64.8 ± 11 J kg^−1^ min^−1^ in the morning session and 81 ± 15 J kg^−1^ min^−1^ in the afternoon session. For both the morning and the afternoon session, RMR values were averaged for the RMR values obtained before each overground and the treadmill walking trial. Although we measured oxygen consumption rate in the afternoon session at least 2 h after finishing lunch, we cannot rule out some remaining thermic effects of food leading to the elevation in the resting oxygen consumption rate. Thus, RMR is a potential source for variability of GCoW, and this strengthens our choice to focus on NCoW. The question may be raised whether our conclusions with respect to NCoW would have been different if we had measured RMR during standing rather than sitting. It is possible that the metabolic cost of standing is higher in OA than in YA due to balance being impaired in OA. In that case, if we had subtracted RMR in standing rather than sitting from the gross metabolic rate of walking, the difference in NCoW between OA and YA would have been even smaller.

## Discussion

When summarizing published data on differences between YA and OA for MCoW, we found OA have a statistically significantly elevated MCoW compared to YA (Das Gupta et al. 2019). However, we could not determine whether this elevation was caused directly by age or was due to confounders and in particular due to walking on a treadmill. The purpose of the present study was to determine to which extent the elevated MCoW reported in the literature for OA is due to the effect of age per se. We solicited matched, healthy, active and fit OA and YA, determined their PWS, and measured their MCoW at that speed both overground and on a treadmill. We found both OA and YA to have a PWS of 1.27 m s^−1^. Only Malatesta et al. (2017) found a similar PWS in OA and YA; in other studies, PWS was lower in OA than in YA [e.g., Jones et al. 2009; Martin et al. 1992; Waters et al. 1983, 1988)]. Note, however, that all but the studies from (Waters et al. 1983, 1988) measured PWS on a treadmill and we cannot rule out that treadmill walking influences PWS. In any case, in our study, differences in anthropometrics and PWS were not present and could be ruled out as confounders, so that we could purely focus on answering the following three questions: (1) is NCoW in overground walking higher in OA than in YA? (2) Is NCoW in treadmill walking higher than in overground walking in OA and in YA? (3) Is NCoW in treadmill walking higher in OA than in YA? Below, we will answer each of these questions, but not before discussing the statistical power of our study.

The question of whether NCoW was higher in overground walking in OA than in YA is obviously the most ecologically relevant question. After all, participating in society requires overground walking, not treadmill walking. Our Mixed ANOVA detected no main effect of group on NCoW, which implies that NCoW was not higher in overground walking in OA than in YA. In principle, this could be a false negative outcome. After all, we had only 10 participants in each group, and the ANOVA formally required no less than 28 family-wise post-hoc corrections, which may mask relevant differences among groups. In our defense of group sizes: most previous studies reporting an elevated MCoW in OA compared to YA have been performed with similarly sized OA and YA groups, we repeated all measurements in the second session, and we focused on NCoW; NCoW has smaller variability and greater reported effect size than GCoW, probably due to a highly variable RMR [see also (Das Gupta et al. 2019)]. With respect to using a Mixed ANOVA: while this is the formal approach, we can also do simple unpaired Student’s *t* test on the difference in NCoW in the morning overground trial, to minimize the chance of a false negative outcome. Note that this statistical test would be standard for studies investigating the MCoW in OA and YA in a single overground trial [as for example done in Waters et al. (1983, 1988)]. In our morning overground walking trial, the difference in NCoW between OA and YA was only 0.08 J kg^−1^ m^−1^ and not statistically significant (2.64 ± 0.38 J kg^−1^ m^−1^ in OA and 2.56 ± 0.79 J kg^−1^ m^−1^ in YA, *p* = 1) in fact, the difference was negligible and no realistic number of participants would have rendered it statistically significant. In the afternoon trial the difference was 0.3 J kg^−1^ m^−1^; this value is close to the difference between OA and YA emanating from our meta-analysis of studies in the literature (~ 0.4 J kg^−1^ m^−1^), but even in a simple unpaired Student’s *t* test it was not statistically significant (*p* = 0.18, Cohen’s *d* = 0.6). Even if there had been a statistically significant difference in the afternoon overground trial, it would be difficult to attribute it to an effect of age per se. Merely looking at trends in the time histories of NCoW in Fig. 3, we see that the difference between OA and YA in NCoW during overground walking had grown in the afternoon relative to the morning session because NCoW of YA has dropped, not because NCoW of OA had increased. Interestingly, GCoW in YA had not changed; the drop in NCoW was due to an unexpected increase in RMR in YA. All in all, we feel safe to conclude that there is no direct effect of age on NCoW at PWS in overground walking.

Even though we did not find a direct effect of age on MCoW, it seemed that walking at PWS was more challenging for OA than for YA. After all, none of our YA felt any fatigue at the end of the day of measurements but, as we already mentioned, some of our OA felt fatigued at the end of the afternoon treadmill trial, even after walking 800 m. We can think of several possible reasons. One is that the rest period we had included between the morning and afternoon sessions and/or that between the overground trial and treadmill trial within a session, was sufficient for YA but not for OA. A second possible reason is that OA overestimated their PWS, wanting to show in the morning overground trial that they were fit. A third possible reason is that OA had slower oxygen uptake kinetics and a lower aerobic capacity than YA. It has been shown in the literature that with age there is a marked reduction in lactate threshold, critical power and the maximal rate of oxygen uptake (Conley et al. 2000; Hawkins and Wiswell 2003; Wolthuis et al. 1977; Rossiter 2011). Slower oxygen uptake kinetics could be related to a lowering of critical power, leading to a continued increase in oxygen consumption during treadmill walking and earlier fatigue (Pooles et al. 1988; Rossiter 2011). Furthermore, we have not measured maximal rate of oxygen uptake in our subjects, but it could well be that having the same NCoW as YA presented a relatively higher exercise intensity for OA than YA in our study. A final reason could also be that OA recruited less efficient muscle fibers leading to fatigue (Barclay 1996; Jones et al. 2011; Woledge 1998) during the treadmill walking sessions.

We believe that the results obtained in our OA participants may be generalized to healthy and fit elderly in general. This is because both our YA and OA were normal community-dwelling humans able to carry out their daily chores independently. None of them were involved in any kind of specific exercise programs, or strength and endurance training. Our results differ from those of the only two previous studies in which NCoW in YA and OA was measured during overground walking (Waters et al. 1983, 1988) and NCoW was found to be higher in OA than in YA by 0.29 and 0.26 J kg^−1^ m^−1^, respectively. However, in those studies, OA were walking at a significantly lower PWS than YA, which we marked as our first confounder. It is known from the literature that MCoW depends on walking speed (Ralston 1958; Zarrugh et al. 1974) and the U-shaped relationship between MCoW and walking speed is of similar shape in YA and OA (Malatesta et al. 2003; Martin et al. 1992; Mian et al. 2006; Sanseverino et al. 2018). Furthermore, as we also know from the literature that YA select a PWS near the minimum MCoW (e.g., Ralston 1958), it may well be that the measured increase in the reported NCoW in OA in the studies of Waters et al. (1983, 1988) was due to OA selecting a lower PWS (a scenario explained schematically in Fig. 1C), as acknowledged by Waters et al. themselves (Waters et al. 1983, 1988).

We asked our second question of whether NCoW in treadmill walking is higher than in overground walking in OA and in YA because, except for the two studies of (Waters et al. 1983, 1988), all studies on the difference in MCoW between OA and YA in the literature were on treadmill walking. It has already been shown that OA have a 23% higher metabolic energy requirement during treadmill walking than during overground walking at PWS (Parvataneni et al. 2009). It may be that OA react differently to treadmill walking (Schellenbach et al. 2010; Wass et al. 2005), causing the use of a treadmill to be a confounder. Our findings support this idea. In YA, no differences in NCoW were found between treadmill and overground. In OA, however, NCoW was higher on the treadmill than overground (Fig. 3, columns 1 and 2). This was already the case after 5 min of treadmill walking, when none of the OA reported any fatigue. Also, in the morning treadmill trial, NCoW seemed to increase until the end in OA (Fig. 3), while we had expected it to reach a steady state just like in YA. In OA, during the last 3 min of the morning treadmill trial, NCoW was substantially elevated by 0.6 J kg^−1^ m^−1^compared to NCoW in the morning overground trial. The trend for NCoW to increase could perhaps be related to fatigue. As a matter of fact, two participants in the OA group specifically indicated that they became fatigued during the trial, and similar accounts during pilots were the reason for making the afternoon treadmill trial shorter. Fatigue could cause participants to change to a different walking pattern requiring more mechanical and hence more metabolic energy. In the afternoon treadmill trial, NCoW in OA ended up somewhat lower than in the morning treadmill trial and the difference would have been statistically significant if we only had the two treadmill trials in OA and had used a paired Student’s *t* test (*p* = 0.02, Cohen’s *d* = 0.86). Perhaps OA had become familiar with treadmill walking and were more relaxed (but note that NCoW was still higher than during the overground trials). Or perhaps NCoW in OA would have increased further if participants had kept on walking as long as they did in the morning treadmill trial. Be that as it may, in the morning session YA did not have a higher NCoW on the treadmill than overground, and OA clearly did have a higher NCoW on the treadmill. Hence, YA and OA did react differently to treadmill walking, and the treadmill may have acted as confounder in the literature on age effects on MCoW, especially when only one treadmill trial was included.

The finding that OA react differently to treadmill walking than YA brings us to the third question of whether NCoW in treadmill walking is higher in OA than in YA. This comparison is between groups, and due to the inter-individual variation the chance of a false negative outcome is larger than in the within-subject comparison made for question 2. In the morning session, NCoW was higher in OA than in YA by 0.66 J kg^−1^ m^−1^. The Mixed ANOVA did not detect this group effect. However, studies on the effects of age on MCoW tend to have only one treadmill trial. Such studies rely on an unpaired Student’s *t* test on the difference in NCoW between OA and YA; when we performed this test on the 0.66 J kg^−1^ m^−1^ difference in treadmill NCoW between OA and YA in the morning session, it was statistically significant (*p* = 0.03, Cohen’s *d* = 1.03). Our data thus suggests that the elevated metabolic cost of walking in OA compared to YA, which is reported in the literature, is not an effect of age per se, but due to an elevated metabolic cost of treadmill walking compared to overground walking in OA but not in YA. In the afternoon session, the difference in NCoW between OA and YA on the treadmill was 0.36 J kg^−1^ m^−1^. This is a smaller difference than in the morning session, yet still close to the difference between OA and YA typically reported in the literature (Das Gupta et al. 2019). As mentioned before, it cannot be ruled out that this difference might have been increased if the afternoon treadmill trial had been longer. In any case, we found that even after our second treadmill session—thus after at least 24 min of treadmill walking—NCoW was not fully restored to values measured overground. In the literature, treadmill trials range from 1.2 min (Pincheira et al. 2017) to 10 min (Ortega and Farley 2015) per walking condition. This may thus well be too short to arrive at values of NCoW that are comparable to those measured overground. Our results indicate that treadmills should be used carefully when studying differences in locomotion between OA and YA. It remains to be established if OA familiarize to treadmill walking after a longer time than used in our study; perhaps they do not familiarize at all.

It would be interesting to understand why OA have a higher metabolic cost at PWS in treadmill walking than in overground walking, while YA do not. Considering that the difference is already present after a few minutes of treadmill walking, it is unlikely to be due to fatigue. During the experiments, we noted that several of the participants in the OA group had difficulty with the task of treadmill walking. Also, some of them spontaneously reported feeling anxious. In this context it is relevant to note that the walking surface of the treadmill that we used was about 60 cm above the floor. Even though there was a platform around the treadmill belt, it is possible that anxiety caused OA to walk with more co-contraction on the treadmill than overground, which in turn caused NCoW to be higher during treadmill walking, as already reported in previous studies (Mian et al. 2006; Peterson and Martin 2010; Pincheira et al. 2017).

## Conclusion

In this study we compared the metabolic cost of walking in healthy, active and anthropometrically matched OA and YA overground and on a treadmill in a morning session and in an afternoon session. PWS was the same in the two groups. Considering that there was only a negligible difference in NCoW between OA and YA in the morning overground trial, it seems safe to conclude that there is no effect of age per se on NCoW at PWS. While NCoW in YA seemed to be the same on a treadmill and overground, it was elevated on the treadmill in OA, and statistically significantly so in the morning session. In the literature, a higher metabolic cost of walking is reported for OA than for YA at the same walking speed on a treadmill. Considering the results of our study, this is likely to be due to a differential reaction of OA and YA to treadmill walking, and not to an effect of age per se on the cost of walking at a given speed.

## Data Availability

The datasets generated during and/or analyzed during the current study are available from the corresponding author on reasonable request.

## Abbreviations

ANCOVA: Analysis of covariance
ANOVA: Analysis of variance
BAI: Body adiposity index
BMI: Body mass index
*d*: Cohen’s *d*
EMG: Electromyography
GCoW: Gross cost of walking
MCoW: Metabolic cost of walking
NCoW: Net cost of walking
OA: Older adults
PWS: Preferred walking speed
RER: Respiratory exchange ratio
RMR: Resting metabolic rate
SD: Standard deviation
SEM: Standard error of mean
$\dot{V}{\text{O}}_{2}$: Oxygen consumption rate
W/H: Waist-to-height ratio
YA: Young adults
